# Supplementary material for: Use of an individual-based model of pneumococcal carriage for planning a randomized trial of a whole-cell vaccine
Source: PLoS Comput Biol. 2018 Oct 1;14(10):e1006333. doi: 10.1371/journal.pcbi.1006333 (PMC6181404; doi:10.1371/journal.pcbi.1006333)
Supplement: S1 Text — Derivation of the lifespan distribution and age-specific contact weights used in the model. (DOCX) [file pcbi.1006333.s001.docx]

Use of an individual-based model of pneumococcal carriage for planning a randomized trial of a whole-cell vaccine

Francisco Y. Cai^1,2*^, Thomas Fussell^1^, Sarah Cobey^3^, Marc Lipsitch^1,2^

^1^ Department of Epidemiology, Harvard T. H. Chan School of Public Health, Boston, MA, USA

^2^ Center for Communicable Disease Dynamics, Harvard T. H. Chan School of Public Health, Boston, MA, USA

^3^ Department of Ecology and Evolution, University of Chicago, Chicago, IL, USA

* Corresponding author

Email: francisco@mail.harvard.edu

# S1 Text. Model age structure

The age distribution and age-specific contact rate of hosts is important to consider in pneumococcal transmission modeling, since carriage prevalence varies with age [1,2], as does frequency of contact with other age groups [3,4].

The age distribution of the simulated hosts was matched to the 2015 age distribution in Kenya, based on data from the United Nations World Population Prospects [5]. The number of simulated hosts was constant, and for a fixed-sized population, we can set its age distribution by choosing the correct lifespan distribution: For a simulated host, the probability of living exactly *n* years is calculated as the difference in the number of *n­*-year old people and *n + 1*-year old people, divided by the total number of people. For this method to be valid, the age distribution must be monotonically decreasing, i.e. there cannot be more people in an older age class as compared to any younger age class. This is the case for Kenya’s age distribution in 2015. The World Population Prospects data was given in 5-year age classes, which we linearly interpolated to obtain 1-year age classes. The oldest age class in the data was 100 years or greater; in our model, we assume that the maximum lifespan is 101 years.

We derived age-specific mixing weights from social contact data collected in Kilifi, Kenya from 2011 to 2012 by Kiti et al [3]. Specifically, normalized the age group-specific average number of contacts per day by the size of the contacting age group and the size of the contacted age group. Since we fit the overall contact rate, for simplicity, we scaled the mixing weights so the maximum is 1. The weights used can be found in **S1 Table**.

# References

1. Abdullahi O, Karani A, Tigoi CC, Mugo D, Kungu S, Wanjiru E, et al. The prevalence and risk factors for pneumococcal colonization of the nasopharynx among children in Kilifi District, Kenya. Ratner AJ, editor. PLoS ONE. Public Library of Science; 2012;7(2):e30787.

2. Gray BM, TURNER ME, Dillon HC. Epidemiologic studies of Streptococcus pneumoniae in infants. The effects of season and age on pneumococcal acquisition and carriage in the first 24 months of life. Am J Epidemiol. Oxford University Press; 1982 Oct;116(4):692–703.

3. Kiti MC, Kinyanjui TM, Koech DC, Munywoki PK, Medley GF, Nokes DJ. Quantifying age-related rates of social contact using diaries in a rural coastal population of Kenya. Borrmann S, editor. PLoS ONE. Public Library of Science; 2014;9(8):e104786.

4. Wallinga J, Teunis P, Kretzschmar M. Using data on social contacts to estimate age-specific transmission parameters for respiratory-spread infectious agents. Am J Epidemiol. Oxford University Press; 2006 Nov 15;164(10):936–44.

5. United Nations, Department of Economic and Social Affairs, Population Division (2015). World Population Prospects: The 2015 Revision, Volume I: Comprehensive Tables.
