# Supplementary material for: Use of an individual-based model of pneumococcal carriage for planning a randomized trial of a whole-cell vaccine
Source: PLoS Comput Biol. 2018 Oct 1;14(10):e1006333. doi: 10.1371/journal.pcbi.1006333 (PMC6181404; doi:10.1371/journal.pcbi.1006333)
Supplement: S2 Text — Mathematical description of the algorithm used to fit the transmission model to carriage prevalence data. (DOCX) [file pcbi.1006333.s002.docx]

Use of an individual-based model of pneumococcal carriage for planning a randomized trial of a whole-cell vaccine

Francisco Y. Cai^1,2*^, Thomas Fussell^1^, Sarah Cobey^3^, Marc Lipsitch^1,2^

^1^ Department of Epidemiology, Harvard T. H. Chan School of Public Health, Boston, MA, USA

^2^ Center for Communicable Disease Dynamics, Harvard T. H. Chan School of Public Health, Boston, MA, USA

^3^ Department of Ecology and Evolution, University of Chicago, Chicago, IL, USA

* Corresponding author

Email: francisco@mail.harvard.edu

# S2 Text. Model fitting algorithm

To simulate specific epidemiological settings, we implemented an algorithm that fit model parameters to given serotype-specific carriage prevalences, e.g. prevalences from survey data. In our model, the prevalence of each serotype is determined primarily by its fitness parameter and the overall contact rate shared by all serotypes. The fitness parameter can take values, possibly non-integral, from 1 to $n_{s}$, the number of serotypes, Lower values correspond to better fitness. Lowering the fitness parameter results in two phenotypic changes—longer colonization duration and enhanced competitive ability—that both increase prevalence. Hence, there is a monotonic relationship between a serotype’s fitness parameter and its expected carriage prevalence, and this allows us to tune the fitness parameters in a straightforward manner.

The algorithm iteratively updates its estimate of the serotype fitness parameters. Let the current estimate at the start of iteration $k$ be denoted by the vector $f^{k}$, indexed by serotype. We run a simulation using $f^{k}$. For serotype $s$, let $\hat{p}_{s}^{k}$ be its average prevalence over the last 25 simulation years, $p_{s}$ be its observed prevalence, and $\delta_{s}^{k}= \hat{p}_{s}^{k}-p_{S}$ be the serotype-specific prevalence error. Based on this error, we update our estimate of the serotype’s fitness parameter according to:

$$\begin{aligned} f_{s}^{k+1}=min\left( n_{s},\max\left( 1, f_{s}^{k}\left( 1+w_{s}^{k}\delta_{s}^{k} \right) \right) \right),\#\left( 1 \right) \end{aligned}$$

where the prevalence error is weighted by a factor $w_{s}^{k}$ (**S2 Fig A**). This factor is also updated iteratively, by comparing the prevalence error between the current and previous iteration. If the magnitude of the prevalence error is not decreasing enough between iterations, we increase the influence of the prevalence error in our updating of the fitness parameter, i.e. if ${\text{sgn}(\delta}_{s}^{k})= \text{sgn}(\delta_{s}^{k-1})$and $\left| \delta_{s}^{k} \right|>K_{T}|\delta_{s}^{k-1}|$, then

$$\begin{aligned} w_{s}^{k+1}= {K_{w}w}_{s}^{k},\#\left( 2 \right) \end{aligned}$$

where $K_{t}$ is a positive constant and $K_{w}$ is a constant greater than 1. On the other hand, if the magnitude of the prevalence error decreased enough between iterations, or if it has changed signs and has become larger in magnitude, then we reduce the influence of the prevalence error in our update, i.e. if ${\text{sgn}(\delta}_{s}^{k})= \text{sgn}(\delta_{s}^{k-1})$and $\left| \delta_{s}^{k} \right|\leq K_{T}|\delta_{s}^{k-1}|$ or ${\text{sgn}(\delta}_{s}^{k})\neq\text{sgn}(\delta_{s}^{k-1})$and $\left| \delta_{s}^{k} \right|>|\delta_{s}^{k-1}|$, then

$$\begin{aligned} w_{s}^{k+1}= {K_{c}w}_{s}^{k},\#\left( 3 \right) \end{aligned}$$

where $K_{c}$ is positive constant less than 1. By adjusting $w_{s}^{k}$ between iterations, we facilitate convergence of the fitness parameters: Equation (2) allows the algorithm to make larger adjustments when it is progressing too slowly, and Equation (3) causes the algorithm to be more cautious it is progressing quickly, or when the simulated prevalences start to oscillate around the observed prevalence. The latter is an indication that we are close to the optimal value for the fitness parameter—since the simulations are stochastic, we would not expect a properly fitted model to reproduce the observed prevalence exactly, but rather a distribution of simulated prevalences centered on the observed prevalence (**S2 Fig B**).

This algorithm attempts to fit all serotype-specific prevalences simultaneously. It assumes that adjusting the fitness parameter of one serotype does not affect the prevalence of another serotype. Since there is competition between serotypes for hosts, that assumption is not strictly true. Nevertheless, we find that in practice, the fitting algorithm is able to converge reasonably quickly, within 125 iterations when using a population size of 20,000.

There are $n_{s}$ observed serotype-specific prevalences we are fitting to, but $n_{s}+1$ parameters: the $n_{s}$ serotype fitness parameters and the overall contact rate. So that the model is not underspecified, we fix the fitness parameter for the fittest serotype to be 1, which corresponds to an intrinsic colonization duration of 150 days and a relative reduction of 0.25 in the risk of colonization by other strains. With one of the fitness parameter fixed, we are free to fit the contact rate. Let $\beta^{k}$ be the current estimate of the contact rate in iteration $k$. Let $p^{k}= \sum_{s} p_{s}^{k}$ be simulated carriage prevalence during iteration $k$, $p= \sum_{s} p_{s}$ be the observed carriage prevalence, and $\delta^{k}= p^{k}-p$be the total prevalence error. The update equation for $\beta$ is similar to that of the fitness parameters:

$$\begin{aligned} \beta^{k+1}=\max\left( 0, \beta^{k}\left( 1-w_{\beta}^{k}\delta^{k} \right) \right),\#\left( 4 \right) \end{aligned}$$

where $w_{\beta}$ is a positive constant. As before $w_{\beta}^{k}$ is updated as well, in the same fashion as described above for $w_{s}^{k}$, but with updating rules based on the $\delta^{k}$ rather than $\delta_{s}^{k}.$ Parameters related to the fitting algorithm are summarized in **S3 Table**.
