# Supplementary material for: Use of an individual-based model of pneumococcal carriage for planning a randomized trial of a whole-cell vaccine
Source: PLoS Comput Biol. 2018 Oct 1;14(10):e1006333. doi: 10.1371/journal.pcbi.1006333 (PMC6181404; doi:10.1371/journal.pcbi.1006333)
Supplement: S1 Table — (DOCX) [file pcbi.1006333.s003.docx]

**S1 Table**. **Age-specific mixing weights**.

| Age (years) | <1 | 1-5 | 6-14 | 15-20 | 21-50 | >50 |
| --- | --- | --- | --- | --- | --- | --- |
| <1 | 0.1391 | 0.3739 | 0.4017 | 0.2938 | 0.4015 | 0.2566 |
| 1-5 | 0.3739 | 0.6283 | 0.5460 | 0.2844 | 0.3561 | 0.2517 |
| 6-14 | 0.4017 | 0.5460 | 0.8344 | 0.4775 | 0.3067 | 0.2304 |
| 15-20 | 0.2938 | 0.2844 | 0.4775 | 1.0000 | 0.4243 | 0.2877 |
| 21-50 | 0.4015 | 0.3561 | 0.3067 | 0.4243 | 0.7304 | 0.5665 |
| >50 | 0.2566 | 0.2517 | 0.2304 | 0.2877 | 0.5665 | 0.5582 |
