# Supplementary material for: Use of an individual-based model of pneumococcal carriage for planning a randomized trial of a whole-cell vaccine
Source: PLoS Comput Biol. 2018 Oct 1;14(10):e1006333. doi: 10.1371/journal.pcbi.1006333 (PMC6181404; doi:10.1371/journal.pcbi.1006333)
Supplement: S2 Table — (DOCX) [file pcbi.1006333.s004.docx]

**S2 Table**. **Fitted serotype fitness parameters**.

| Serotype | Parameter value |
| --- | --- |
| 19F | 1.00 |
| 6A | 1.75 |
| 6B | 3.18 |
| 23F | 6.20 |
| 11A | 7.92 |
| 14 | 8.37 |
| 35B | 8.40 |
| 23B | 10.01 |
| 10A | 11.67 |
| 15B | 12.03 |
| 9V | 12.52 |
| 19A | 12.56 |
| 15A | 12.70 |
| 13 | 12.81 |
| 15C | 14.39 |
| 34 | 15.66 |
| 16F | 16.83 |
| 3 | 16.83 |
| 18C | 18.50 |
| 19B | 20.01 |
| 7C | 21.42 |
| 20 | 22.14 |
| 21 | 24.31 |
| 23A | 24.51 |
| 35A | 27.62 |
| 33B | 29.27 |
| 1 | 29.55 |
| 4 | 31.08 |
| 38 | 37.22 |
| 35F | 39.19 |
| 10F | 41.82 |
| 24F | 47.45 |
| 12F | 48.24 |
| 33D | 48.33 |
| 22A | 51.21 |
| 18F | 51.56 |
| 29 | 52.07 |
| 22F | 52.54 |
| 28F | 53.03 |
| 17F | 53.20 |
| 10B | 53.89 |
| 28A | 55.99 |
| 8 | 55.99 |
| 9L | 56.00 |
| 15F | 56.00 |
| 40 | 56.00 |
| 12B | 56.00 |
| 11D | 56.00 |
| 18B | 56.00 |
| 19C | 56.00 |
| 31 | 56.00 |
| 33C | 56.00 |
| 5 | 56.00 |
| 7F | 56.00 |
| 9A | 56.00 |
| 9N | 56.00 |
