# Supplementary material for: Use of an individual-based model of pneumococcal carriage for planning a randomized trial of a whole-cell vaccine
Source: PLoS Comput Biol. 2018 Oct 1;14(10):e1006333. doi: 10.1371/journal.pcbi.1006333 (PMC6181404; doi:10.1371/journal.pcbi.1006333)
Supplement: S3 Table — (DOCX) [file pcbi.1006333.s005.docx]

**S3 Table**. **Parameters of the fitting algorithm**.

| **Symbol** | **Description** | **Value** |
| --- | --- | --- |
| $\beta^{0}$ | Initial overall contact rate | 0.1 |
| $f_{s}^{0}$ | Initial fitness parameter for serotype $s$ | $min(n_{S}, r_{s}+5)$^†^ |
| $w_{\beta}^{0}$ | Relative step size for updating $\beta^{0}$ | 1 |
| $f_{s}^{0}$ | Relative step size for updating $f_{s}^{0}$ | 5 |
| $K_{T}$ | Relative error threshold for reducing relative step size | 0.8 |
| $K_{W}$ | Relative step size expansion factor | 1.05 |
| $K_{C}$ | Relative step size reduction factor | 0.95 |
| - | Years sampled from end of simulation | 25 |
| - | Simulation population size (in thousands) | 20 |

^†^$n_{s}$ is the number of serotypes (56) and $r_{S}$ is the rank of serotype $s$ by observed prevalence.
