# Supplementary figures and images for: Use of an individual-based model of pneumococcal carriage for planning a randomized trial of a whole-cell vaccine
Source: PLoS Comput Biol. 2018 Oct 1;14(10):e1006333. doi: 10.1371/journal.pcbi.1006333 (PMC6181404; doi:10.1371/journal.pcbi.1006333)

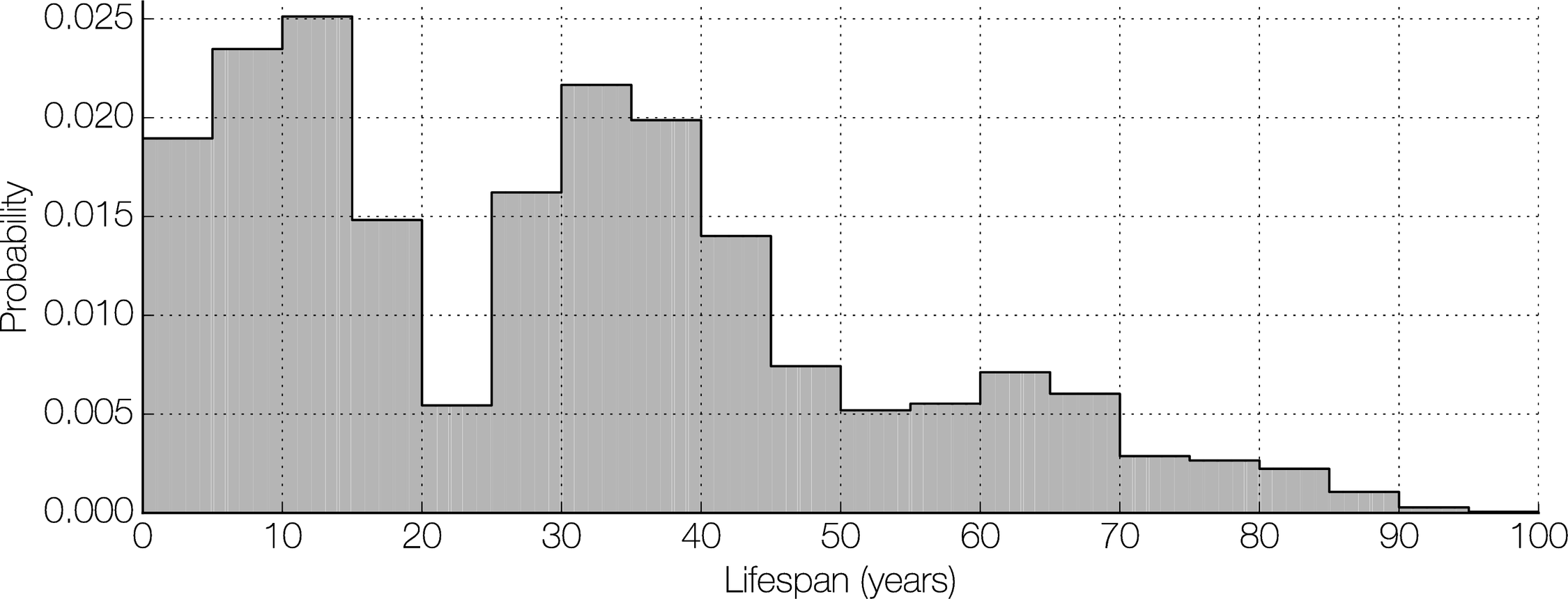

Supplement: S1 Fig — The lifespan distribution used in all simulations. The probabilities refer to 1-year age bins. It is derived by assuming that the 2015 Kenya age distribution [38] is stable, i.e. no population growth. The step-wise nature of the distribution reflects the five-year intervals in the age distribution data. (TIF) [file pcbi.1006333.s006.tif]

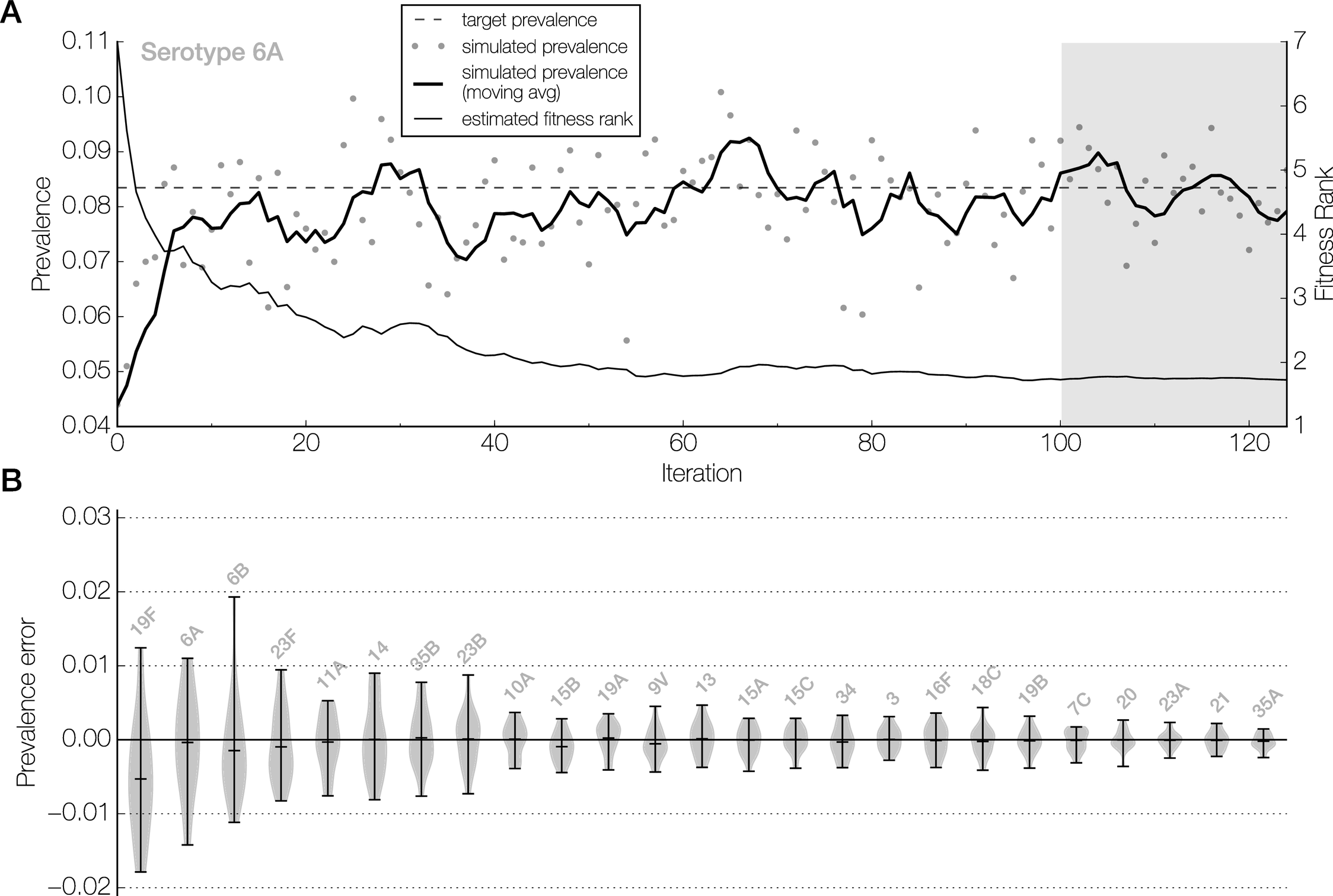

Supplement: S2 Fig — (A) The fitting process for one representative serotype, 6A. The evolving estimate of 6A’s fitness parameter (thin line, right y-axis) and 6A’s simulated prevalence (gray dots, left y-axis) is shown over the course of 125 iterations. Lower values of the fitness parameter correspond to a fitter phenotype. The moving average (thick line, n = 5) of the simulated prevalences more clearly shows the trend of the simulated prevalences towards the target prevalence (horizontal dashed line). The light gray shaded region highlights the last 25 iterations, whose results are considered in (B). (B) One method of assessing the quality of the model fit. The distribution of prevalence errors (simulated minus target prevalence) in the last 25 iterations of the fitting process is shown for the top 25 serotypes (out of 56 total) by target prevalence (ranging from 9.96% for 19F to 0.53% for 35A). Each distribution is represented by a violin plot labeled by serotype name, and with horizontal bars marking the minimum, mean, and maximum values. (TIF) [file pcbi.1006333.s007.tif]

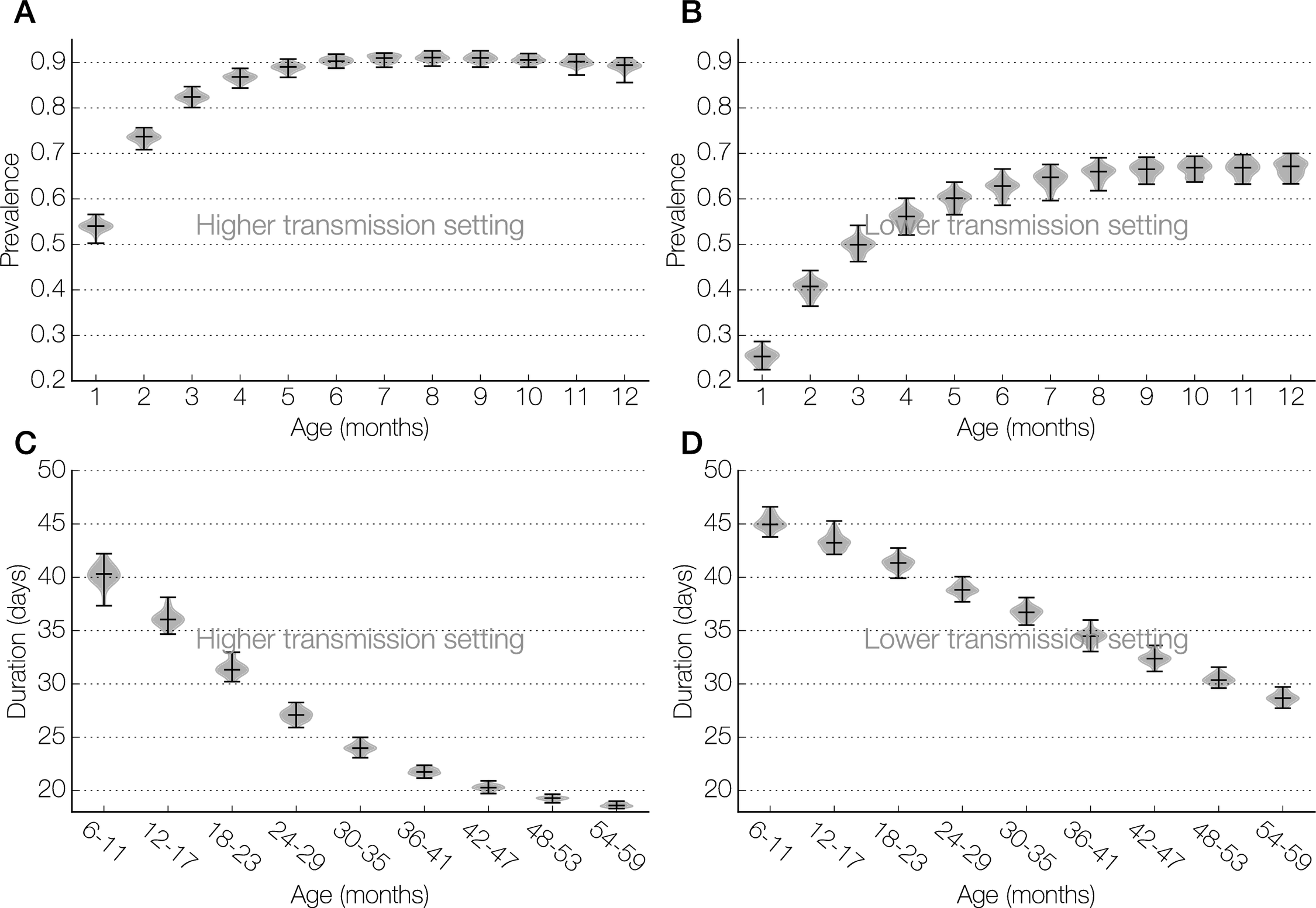

Supplement: S3 Fig — (A, B) Distribution of carriage prevalence in infants, by 1-month age categories, for the higher (A) and lower (B) transmission settings. (C, D) Distribution of carriage duration in infants and toddlers, by 6-month age categories, for the higher (C) and lower (D) transmission settings. Distributions are shown as violin plots, with horizontal bars indicating the minimum, median, and maximum values. (TIF) [file pcbi.1006333.s008.tif]

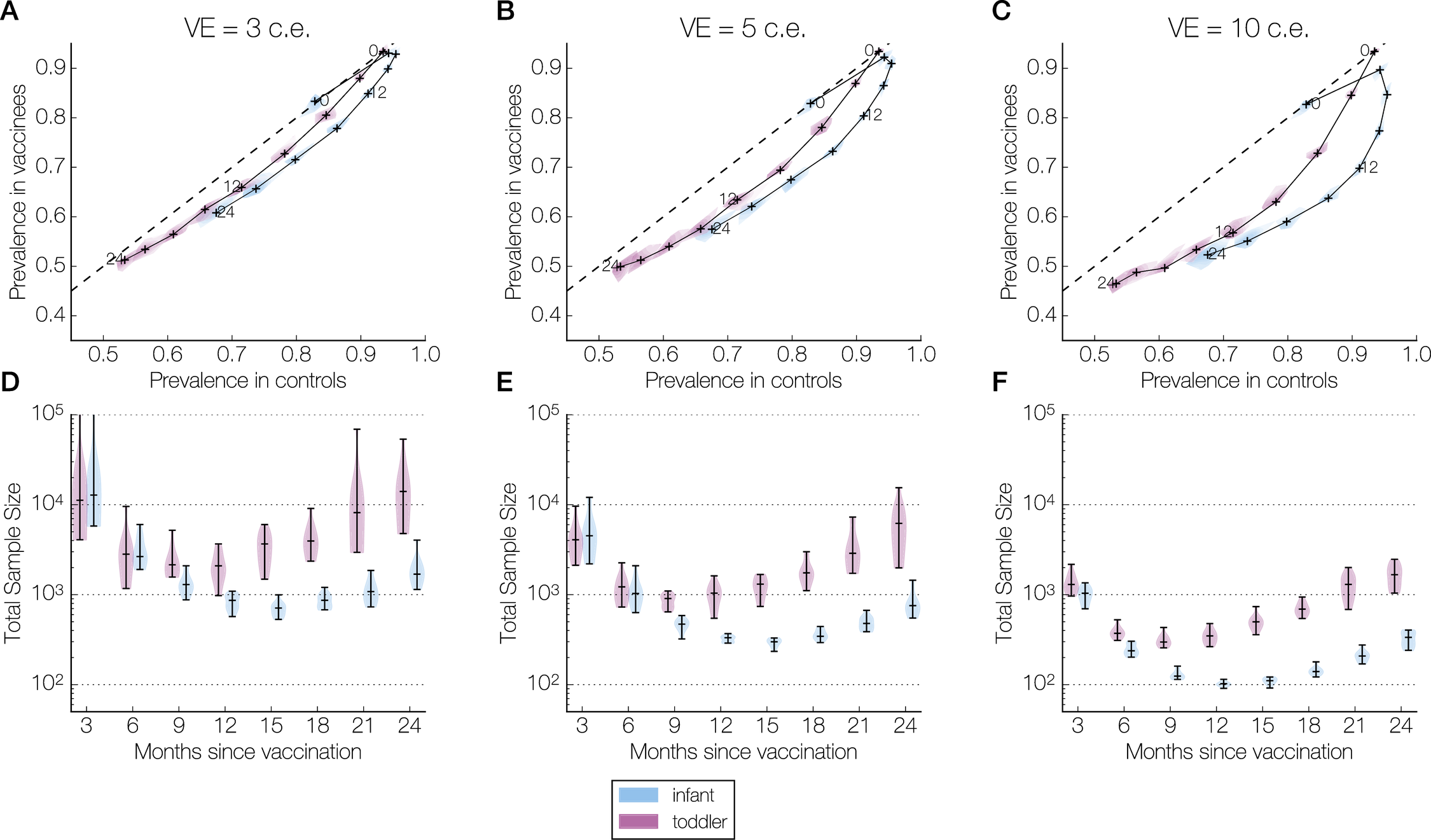

Supplement: S4 Fig — Panels are organized column-wise by wSP vaccine efficacy: 3 colonization equivalents (c.e.), or 53% reduction in carriage duration (A, D); 5 c.e., or 71% (B, E); and 10 c.e., 92% (C, F). Within each panel, results are presented separately for infants (blue) and toddlers (purple). (A-C) The joint kernel density estimate (see Methods) of the control and vaccine arm prevalences at each sampling time (every 3 months until 24 months post-vaccination) is shown as a contour map truncated by the convex hull of the simulated points, with the median values marked by a cross. These crosses are connected chronologically, and those corresponding to 0, 12, and 24 months post-vaccination are labeled. The dashed line indicates equal prevalences in the two arms. (D-F) The kernel density estimate of the total sample size (combined size of both samples) needed to detect a difference between control and vaccine arm prevalences at each sampling time (assuming 80% power, 5% type I error rate, balanced arms). The horizontal bars in each violin plot indicate the minimum, median, and maximum values across all simulations. In (D), the maximum sample sizes for infants and for toddlers at 3 months post-vaccination are greater than one hundred thousand (108 thousand and 105 thousand, respectively) and outside the limits of the y-axis. (TIF) [file pcbi.1006333.s009.tif]

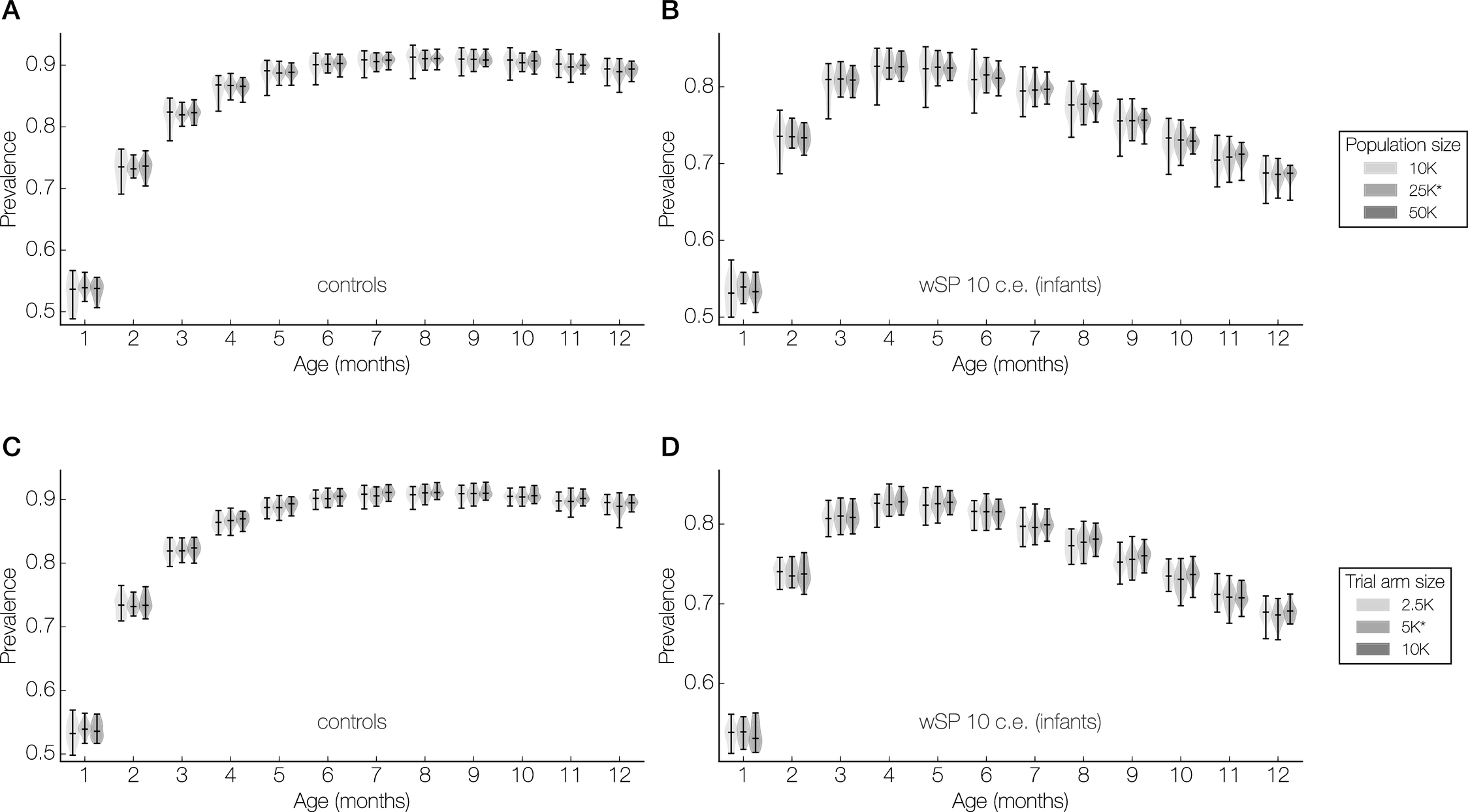

Supplement: S5 Fig — (A, B) The age-specific prevalence in the control (A) and wSP 10 c.e. (conferring an additional 92% reduction in carriage duration; B) infant arms for three different population sizes– 10K, 25K, and 50K individuals–with the trial arm size fixed at 5K participants. (C, D) The age-specific prevalence in the control (C) and wSP 10 c.e. (D) infant arms for three different trial arm sizes– 2.5K, 5K, and 10K participants–with the population size fixed at 25K. Each violin plot shows the distribution of prevalences across 25 simulations, with horizontal bars marking the minimum, median, and maximum values, and darker shades indicating larger population or trial arm sizes. The values used in the main analyses–a population size of 10K and a trial arm size of 5K –are marked with asterisks in the legends. (TIF) [file pcbi.1006333.s010.tif]
